# Prevalence and molecular characteristics of *Shewanella* infection in diarrhea patients in Beijing, China 2017–2019

**DOI:** 10.3389/fmicb.2024.1293577

**Published:** 2024-01-31

**Authors:** Ying Kang, Keyi Yu, Zhenzhou Huang, Bo Pang, Shengtian Liu, Tao Peng, Ying Li, Duochun Wang

**Affiliations:** ^1^Shunyi District Center for Disease Control and Prevention, Beijing, China; ^2^Workstation for Microbial Infectious Disease, Shunyi District Center for Disease Control and Prevention, Beijing, China; ^3^National Key Laboratory of Intelligent Tracking and Forecasting for Infectious Diseases, National Institute for Communicable Disease Control and Prevention, Chinese Center for Disease Control and Prevention (China CDC), Beijing, China; ^4^Hangzhou Center for Disease Control and Prevention, Hangzhou, Zhejiang, China

**Keywords:** *Shewanella*, prevalence, molecular characteristics, diarrhea, antibiotic susceptibility

## Abstract

**Introduction:**

*Shewanella* is an important opportunistic pathogen distributed in marine environments that has caused an increasing number of clinical infections. However, there are few reports on the distribution and characteristics of *Shewanella* in the diarrheal pathogen spectrum. In this study, we have systematically described the prevalence of *Shewanella* infections in diarrhea patients in Beijing, China 2017–2019, and genome characteristics and antimicrobial susceptibility of *Shewanella* isolates.

**Methods:**

Stool samples were collected from diarrhea patients in a surveillance project from 2017 to 2019. *Shewanella* strains were isolated, and identified using VITEKR 2 COMPACT and MALDI-TOF MS. Average nucleotide identity (ANI) analysis, multi-locus sequence typing (MLST), phylogenetic analysis, virulence-associated genes and antimicrobial resistance genes analysis were used for genome characteristics description. The antibiotic susceptibility test was performed with microbroth dilution method.

**Results:**

1104 fecal samples were collected, and the *Shewanella* detection rate was 2.36% (26/1104). The main manifestations of infection caused by *Shewanella* spp. were diarrhea (100%, 26/26), abdominal pain (65.38%, 17/26), and vomiting (38.46%, 10/26). The 26 isolates were classified into 3 species (*S. algae* (*n* = 18), *S. indica* (*n* = 5), and *S. chilikensis* (*n* = 3)) and 22 sequence types. Core genome single nucleotide polymorphism-based evolutionary tree identified three clone groups corresponding to three infection events in the same months in 2017 and 2019. The putative virulence-associated gene pool consisted of 56 potential virulence genes, including 19 virulence gene factors. The resistance rates of the 26 isolates to 17 antibiotics from high to low were as follows: polymyxin E (76.92%), cefotaxime (57.69%), ampicillin (50%), ampicillin-sulbactam (34.62%), nalidixic acid (15.38%), ciprofloxacin (11.54%), selectrin (3.846%,1/26), and tetracycline (3.846%, 1/26). The rate of multidrug resistance was 38.46% (10/26).

**Discussion:**

Monitoring for *Shewanella* spp. should be added to the routine surveillance of infectious diarrhea during the epidemic season.

## 1 Introduction

*Shewanella* spp. are a group of gram-negative, oxidase-positive, facultatively anaerobic bacteria distributed in marine environments and the digestive tracts of marine animals. They can also be found in extreme environments with low temperature, high pressure, and high salinity (Kouzuma et al., 2015). Clinical cases related to *Shewanella*, which are opportunistic pathogens, have been frequently reported (Yousfi et al., 2017a). Some *Shewanella* species, such as *S. algae* (Chia-Wei et al., 2022), *S. putrefaciens* (Holt et al., 2005), and *S. xiamenensis* (Zong, 2011; Antonelli et al., 2015), are directly associated with clinical infections. Furthermore, *Shewanella* often causes skin and soft tissue infections, ear, nose, and throat-related diseases, chest and abdominal cavity infections, blood infections, and even cardiovascular and central nervous system diseases (Yu et al., 2022), causing widespread concern both domestically and internationally. Such bacteria have been isolated from clinical samples of patients with diarrhea in recent years (Yiallouros et al., 2013; Dey et al., 2015; Fang et al., 2017). Yonglu et al. (2009) isolated them for the first time from patients with food poisoning. Therefore, diarrheal diseases caused by *Shewanella* spp. require more attention.

However, systematic taxonomic studies of the *Shewanella* genus are not yet complete. Traditional classification and identification methods based on bacterial morphology, cultural characteristics, and biochemical results are difficult to apply to the genus *Shewanella*, because the phenotypic characteristics of its species are very similar (Liu et al., 2013). In addition, it is difficult to distinguish genetically closely related *Shewanella* species through homology analysis of the 16S rRNA gene sequence (Yarza et al., 2014). Molecular typing techniques, such as multilocus sequence typing (MLST), have been applied for surveillance of the molecular epidemiology of *Shewanella* (Thorell et al., 2019). Whole-genome sequencing can be used to quickly determine the genetic and evolutionary characteristics of *Shewanella*. Moreover, whole-genome sequencing has good sensitivity and specificity for predicting drug resistance and virulence-related genes (Lee et al., 2016).

However, relevant studies on the distribution and characteristics of *Shewanella* in the diarrheal pathogen spectrum in China are still lacking. In the present study, we used whole-genome sequencing and bioinformatics tools for an in-depth analysis of epidemic and pathogenic characteristics based on monitoring the diarrheal pathogen in Beijing, China, from 2017 to 2019.

## 2 Materials and methods

### 2.1 Sample collection

According to the guidelines of the local foodborne disease surveillance project in Beijing, patients with diarrhea enrolled in this study were outpatients who presented with acute diarrhea. This was defined as ≥3 passages of watery, loose, mucus, or bloody stools during a 24 h period in two clinics in Shunyi district, Beijing from January 1st, 2017 to December 30th, 2019. Age, sex, occupation, clinical symptoms, and other information of the patients with diarrhea were collected, and 5 g of fresh stool samples were collected from each patient. The samples were stocked in Cary-Blair medium at 4°C and transported to the laboratory for bacterial isolation within 24 h to isolate and culture *Shewanella*.

### 2.2 *Shewanella* isolation

Fecal samples from the patients were isolated and cultured immediately after delivery to the laboratory. Briefly, a 200 mg stool sample was inoculated into 3% NaCl alkaline peptone water and enriched at 37°C for 24 h. After selective enrichment, a loop of the culture was streaked on TCBS and CHROMagar *Vibrio* color red medium and incubated at 37°C for 24 h. Five presumptive *Shewanella* colonies on the selective agar plate (medium-sized, smooth, raised, rounded colonies with colorless edges and black centers on TCBS agar plates and round, translucent, smooth-surfaced pink-purple colonies on CHROMagar *Vibrio* color red medium) were inoculated in 3% NaCl tryptic soy agar and incubated at 37°C for 24 h. The pure culture of each colony was identified using VITEKR 2 COMPACT (BioMerieux) and MALDI-TOF MS (Bruker).

### 2.3 Whole-genome sequencing and bioinformatics analysis

Genomic DNA was extracted using a Wizard Genomic DNA Extraction Kit (Promega, Madison, WI, USA) following the manufacturer’s instructions. DNA samples were sent to the Beijing Genomics Institution for next-generation sequencing, requiring a total amount > 20 μg. The concentration reached 50 ng/μL and OD260/280 of 1.8–2.0 for individual samples. Low-quality reads were discarded, and clean data were assembled using SOAP *de novo* (version 2.04). After removing contigs with less than 500 bp, QUAST (version 5.0.1) was applied to evaluate the quality of assembled genomes (Gurevich et al., 2013). Prokka (version 1.12) (Seemann, 2014), Prodigal (version 2.6.3) (Hyatt et al., 2010), and RAST were used to annotate genomes.^1^

The genome sequences of 26 laboratory isolates and four type strains (*S. algae* JCM 21037^T^, *S. chilikensis* KCTC 22540^T^, *S. indica* KCTC 23171^T^, and *S. carassii* 08MAS2251^T^) were included in this study. Average nucleotide identity (ANI) analysis was used to evaluate the evolutionary distance between bacteria at the genomic level based on a Perl script as previously described (Huang et al., 2022). ANI values >95% were considered indicative of the same species. MLST was performed using seven housekeeping genes (16S rRNA, *gyrA*, *gyrB*, *infB*, *recN*, *rpoA*, and *topA*) (Huang et al., 2023). Housekeeping gene sequences were extracted from the genomes to obtain the allele numbers and sequence type (ST). BioNumerics 7.1 was used to analyze allele profiles and construct a minimum spanning tree. Prokka (version 1.12) and Roary pan-genome pipeline with an identity cut-off of 95% were used for gene annotation and pan genome analysis, respectively. Snippy was used to refer to core genome single nucleotide polymorphisms, with the genome sequence of *S. algae* JCM 21037^T^ used as a reference. Gubbins was used to remove recombinant sequence sites. The phylogenetic evolutionary tree was constructed using IQ-TREE based on the decombined core genome single nucleotide polymorphisms (maximum likelihood estimation, bootstraps 1,000). In the Virulence Factor Database (VFDB) database, owing to the lack of a virulence factor library related to the *Shewanella* genus, we used *Vibrio* species as a reference for virulence gene analysis because of their close phylogenetic relationship. Potential antimicrobial resistance genes were predicted by Comprehensive Antibiotic Research Database (CARD) (http://arpcard.mcmaster.ca).

### 2.4 Antimicrobial susceptibility testing (AST) for *Shewanella*

An AST panel for aerobic Gram-negative bacilli (Shanghai Fosun Long March Medical Science Co., Ltd., China) was performed using the microbroth dilution method. As there were no breakpoints for *Shewanella*, the results for susceptibility (S), intermediate (I), and drug resistance (R) were interpreted according to the *Enterobacteriaceae* standards of the American Committee for Clinical Laboratory Standardization (Clinical and Laboratory Standards Institute, 2019). *Escherichia coli* ATCC 25922 was used as a control for ampicillin, ampicillin-salbactam, tetracycline, meropenem, polymyxin E, ertapenem, ceftazidime/avibactam, tigecycline, cefotaxime, ceftazidime-avibactam, ciprofloxacin, azithromycin, chloramphenicol, nalidixic acid, streptomycin, selectrin, and amikacin.

### 2.5 Statistical analysis

All statistical analyses were performed using the Stata software version 12.0. The chi-squared test was used to compare the isolation ratios of different pathogens in different months. The *Shewanella* infection ratios between different sexes, among different age groups, and contamination in different suspected food groups were also analyzed using the chi-squared test. Statistical significance was set at *p* < 0.05.

### 2.6 Ethics statement

All aspects of the study were performed in accordance with the national ethics regulations and were approved by the Ethics Committee of the Shunyi District CDC, China. Participants received information on the purpose of the study and their right to keep their information confidential. Written informed consent was obtained from each participant or their parents/guardians.

## 3 Results

### 3.1 Prevalence and clinical characteristics of *Shewanella* infection

In this surveillance study, 1,104 fecal samples were collected. The detection rate of *Shewanella* was 2.36% (26/1104). The detection rates in 2017, 2018, and 2019 were 2.43% (9/371), 2.67% (10/374), and 1.95% (7/359), respectively (Table 1). The major clinical symptoms reported in infectious cases caused by *Shewanella* spp. were diarrhea (100%, 26/26), abdominal pain (65.38%, 17/26), and vomiting (38.46%, 10/26) (Table 1). Each of the 26 patients had diarrhea more than three times per day; among them, six had loose stools (23.08%, 6/26), and the remaining had liquid stools (76.92%, 20/26). The *Shewanella* infection rates in male and female patients were 2.17% (14/644) and 2.61% (12/460), respectively, with no significant difference between the infection rate and sex (*p* = 0.639). The infection rate of *Shewanella* in the 61–80-year-old group ranked first at 2.99% (4/134), but there was no significant difference in *Shewanella* infection among the different age groups (*p* = 0.745, Fisher’s exact test) (Table 2). The three occupations with the highest *Shewanella* infection ratios were commercial service personnel, workers, and household workers, with infection ratios of 4.11% (3/73), 2.82% (6/213), and 2.71% (6/221), respectively. Again, there was no significant difference in *Shewanella* infection among the different occupational groups (*p* = 0.950) (Table 2).

**Table 1:** Prevalence of *Shewanella* infections in diarrhea patients in S District, Beijing, 2017–2019.

Year of specimen collection	No. of stool specimen tested	No. of *Shewanella* positive cases (%)	No. of mixed infection cases (%)	Clinical symptom		
				Diarrhea (%)	Abdominal pain (%)	Vomiting (%)
2017	371	9 (2.43)	3 (0.81)	9 (100)	5 (55.56)	4 (44.44)
2018	374	10 (2.67)	4 (1.07)	10 (100)	7 (70)	4 (40)
2019	359	7 (1.95)	3 (0.84)	7 (100)	5 (71.43)	2 (28.57)
Total	1,104	26 (2.36)	10 (0.91)	26 (100)	17 (65.38)	10 (38.46)

**Table 2:** The detection rate of *Shewanella* in different populations in S District, Beijing, 2017–2019.

	No. of positive cases	No. of cases	Detection rate (%)	χ^2^	*p*
Gender				0.221	0.639
Male	14	644	2.17		
Female	12	460	2.67		
Age				1.857	0.745
≤20	0	70	0		
21 ~ 40	17	667	2.55		
41 ~ 60	5	224	2.23		
61 ~ 80	4	134	2.99		
≥81	0	9	0		
occupation				4.923	0.95
Officials	4	217	1.84		
Workers	6	213	2.82		
Job-waiting people	6	221	2.71		
Retired people	1	47	2.13		
Farmer	3	112	2.68		
Commercial service	3	73	4.11		
Peasant-workers	0	10	0		
Catering services	0	5	0		
Teachers	0	31	0		
Students	0	63	0		
Children	0	9	0		
Medical staff	0	7	0		
Others	3	96	3.13		

### 3.2 Identification of *Shewanella* species

**Figure 1:**
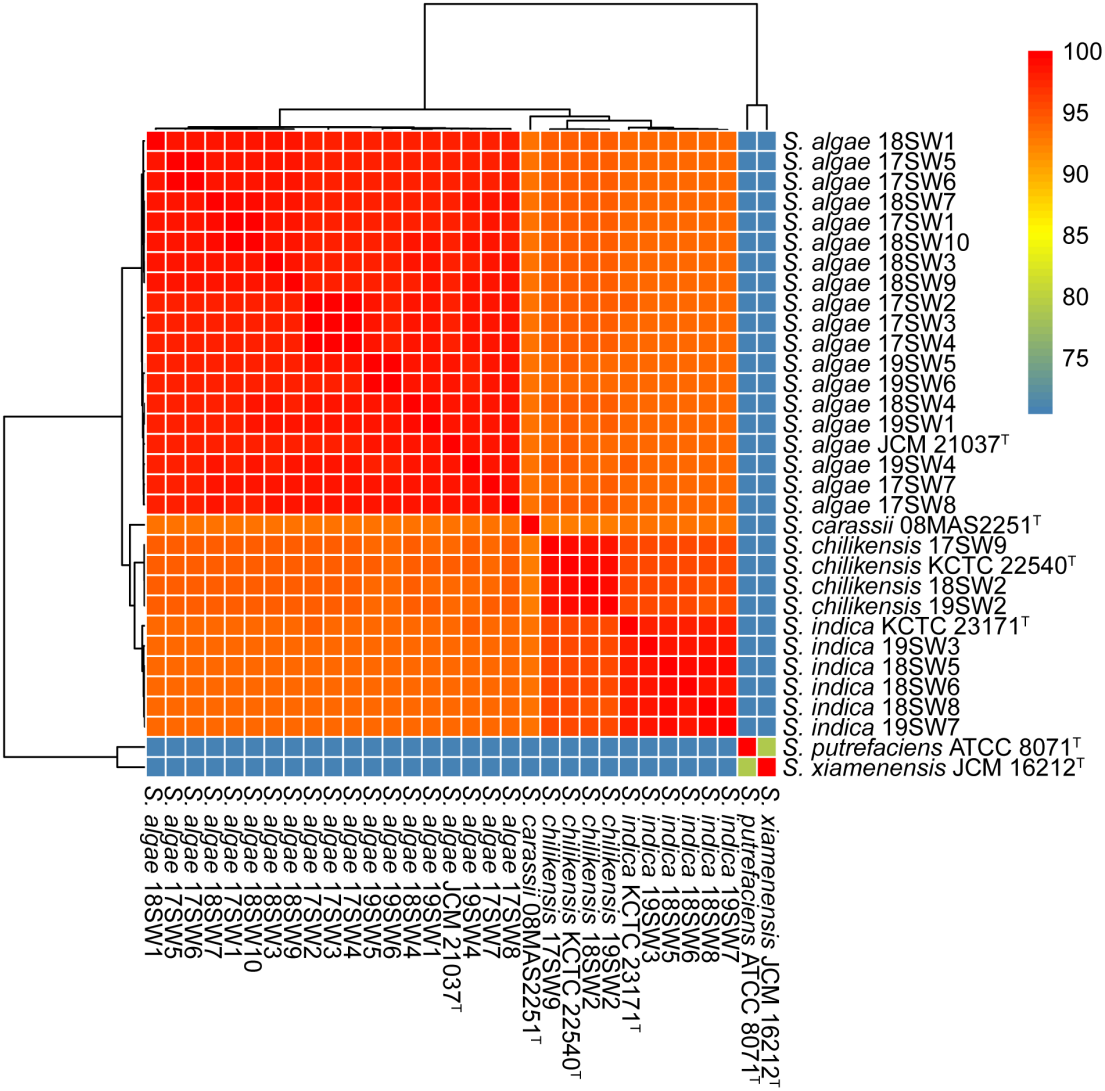
Average nucleotide identity (ANI) values for the species identification for the 26 *Shewanella* strains. Six type strains (*S. algae* JCM 21037^T^ *S. carassii* 08MAS2251^T^ *S. chilikensis* KCTC 22540^T^ *S. indica* KCTC 23171^T^ *S. putrefaciens* ATCC 8071^T^ *S. xiamenensis* JCM 16212^T^) were used as reference genome. The color from blue to red represents the value of ANI from <75 to 100.

The quality and complpeteness of the genomes was shown in the Supplementary Table S1. The ANI heatmap revealed identified 26 *Shewanella* strains (Figure 1). These strains were classified into three distinct species, namely *S. algae* (*n* = 18, 69.23%), *S. indica* (*n* = 5, 19.23%), and *S. chilikensis* (*n* = 3, 11.54%). Both *S. algae* and *S. chilikensis* were detected in 2017–2019, whereas *S. indica* was detected only in 2018–2019.

### 3.3 MLST for *Shewanella*

**Figure 2:**
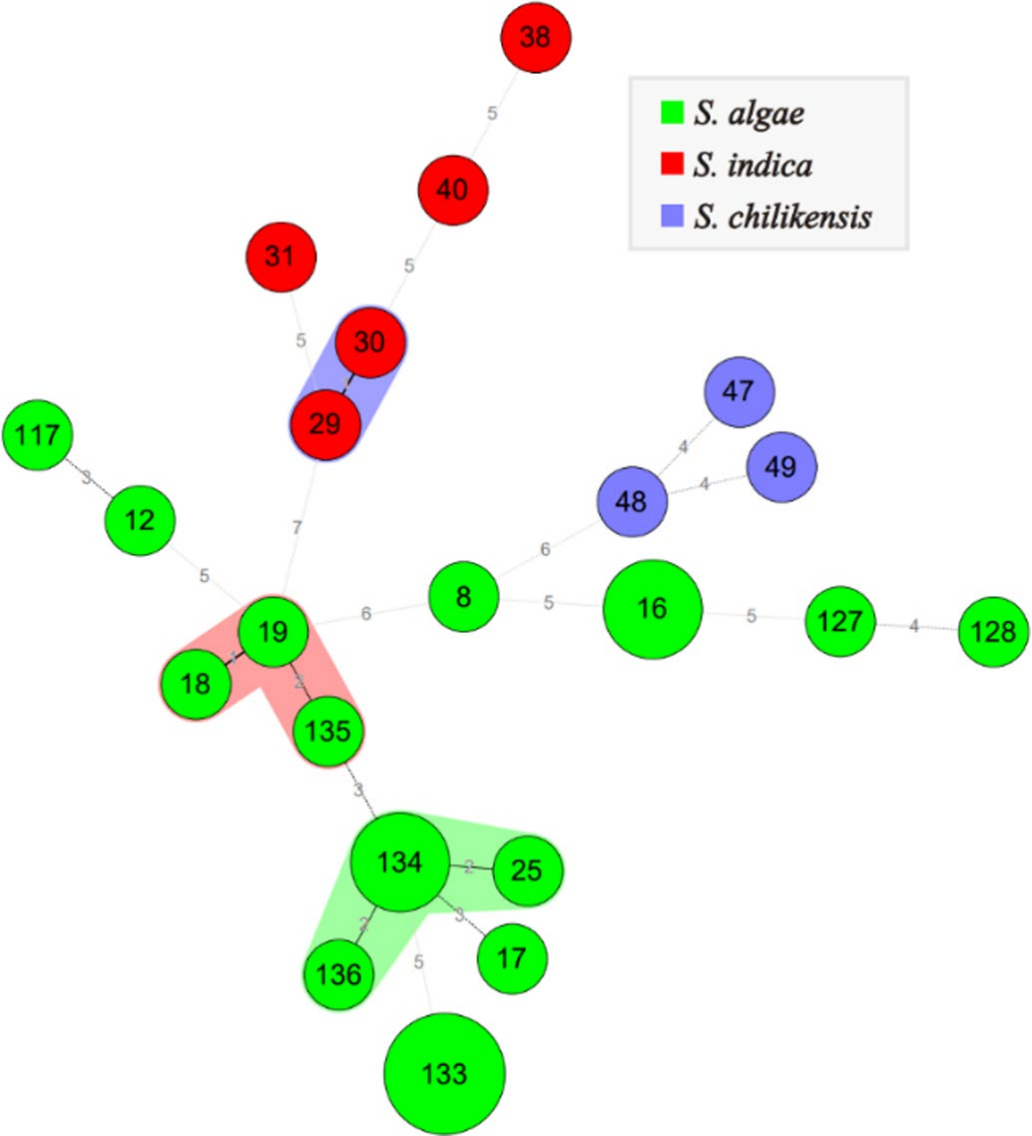
MLST for 26 *Shewanella* strains. Green, red and blue color represent *S. algae*, *S. indica* and *S. chilikensis*, respectively. The size of the circle represents the number of strains.

The allelic profiles of *Shewanella* isolates were classified into 22 STs. Among these, *S. algae* were classified into 16 STs, accounting for 66.7%. *S. indica* and *S. chilikensis* were classified into 5 and 3 STs, accounting for 8.8 and 7.4%, respectively. The minimum spanning tree of different *Shewanella* isolates classified by species, isolation region, and isolation source is shown in Figure 2. No significant regional or isolation source clustering was observed, suggesting high genetic diversity among these strains.

### 3.4 Genomic evolutionary characteristics of *Shewanella*

**Figure 3:**
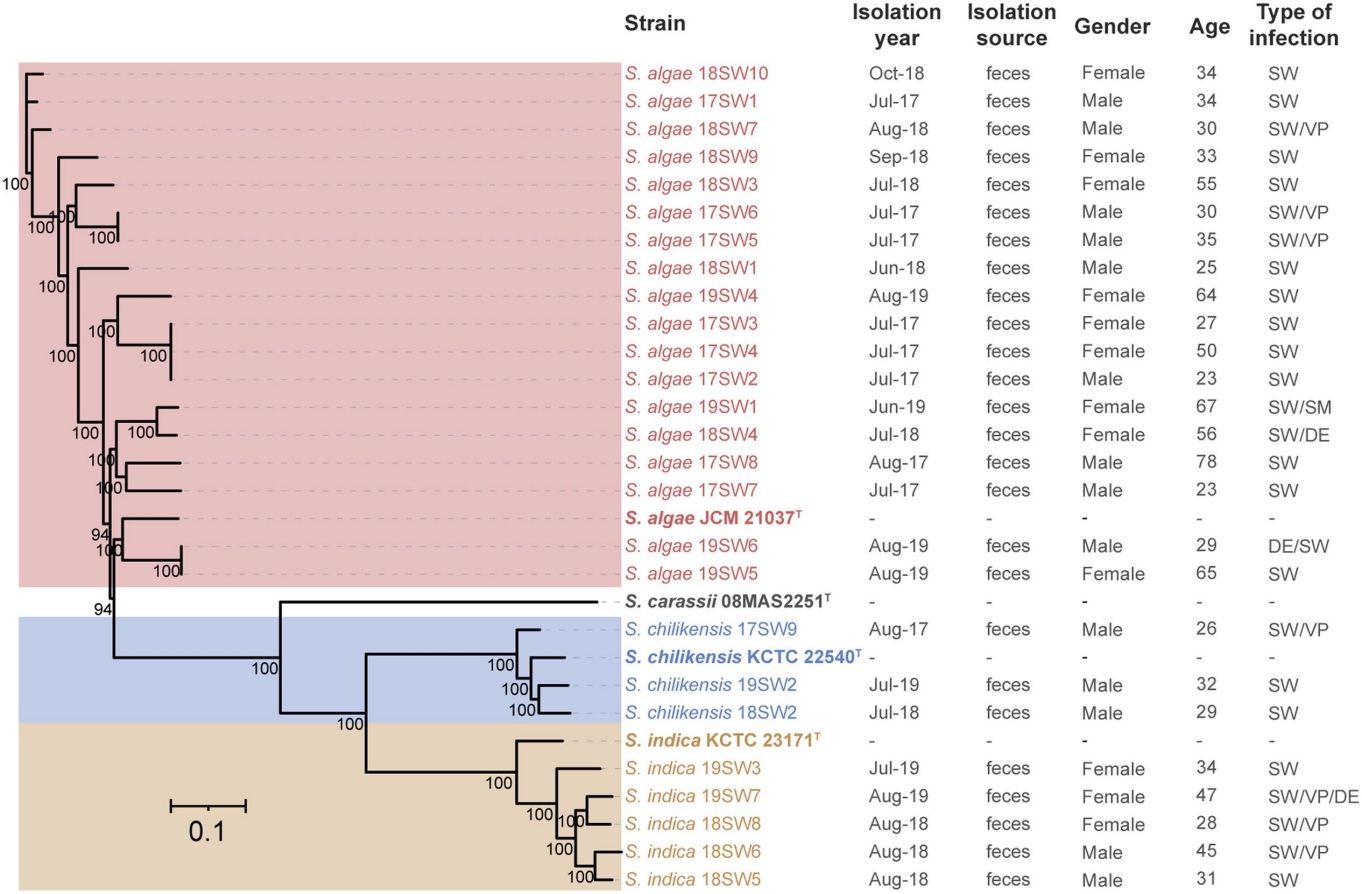
Phylogenetic tree of 26 *Shewanella* isolates and four type strains based on cgSNPs by the maximum-likelihood method.

A total of 301,356 cg of single nucleotide polymorphisms were identified. A phylogenetic tree was constructed using maximum likelihood (Figure 3). Three clone groups were identified among them, namely strains 17SW2/3/4, 17SW5/6, and 19SW5/6, corresponding to three infection events in the same months in 2017 and 2019. However, different clones were detected even in the same month, such as strains 17SW2/3/4, 17SW5/6, 17SW1, and 17SW7, which were isolated in July 2017. Different *Shewanella* species were also detected during the same period: *S. algae* 17SW8 and *S. chilikensis* 17SW9 were detected in August 2017, and *S. algae* 19SW1, *S. chilikensis* 19SW2, and *S. indica* 19SW3 were detected in June 2019. According to statistical analysis, the positive ratio between *S. algae* and other *Shewanella* spp. showed no significant difference in terms of sex, age, or mixed infections.

### 3.5 Distribution of virulence-associated genes

**Figure 4:**
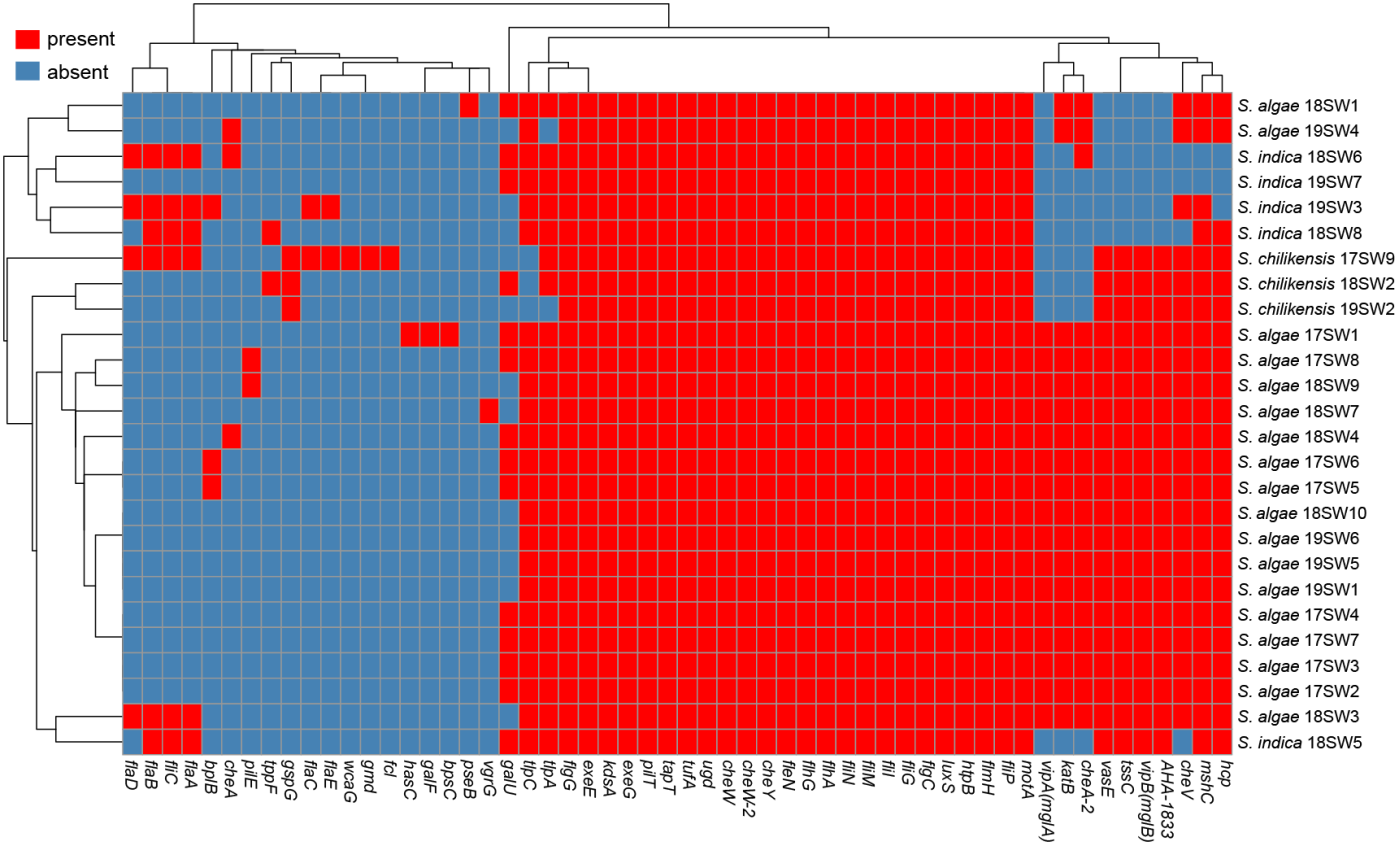
The heatmap of the virulence genes in 26 *Shewanella* strains. Red and blue colors represent the presence and absence of virulence genes, respectively.

The putative virulence-associated gene pool comprised 56 potential virulence genes, including 19 virulence gene factors (Figure 4). Different *Shewanella* species exhibited specific virulence gene patterns. Most strains carried potential virulence genes related to flagella and chemotactic proteins, but rarely carried genes of the III and I secretion systems. Most *S. algae* carried potential virulence genes related to the VI secretion system, including the *hcp* gene related to the inner tube and the effector protein *vgrG*, suggesting that T6SS may be an important virulence factor in *S. algae*. *S. indica* lacked the potential virulence genes related to the VI secretion system, but most strains carried genes related to the flagella. Most *S. chilikensis* strains also harbored potential virulence genes related to the flagella.

### 3.6 Antimicrobial resistance genes analysis

**Figure 5:**
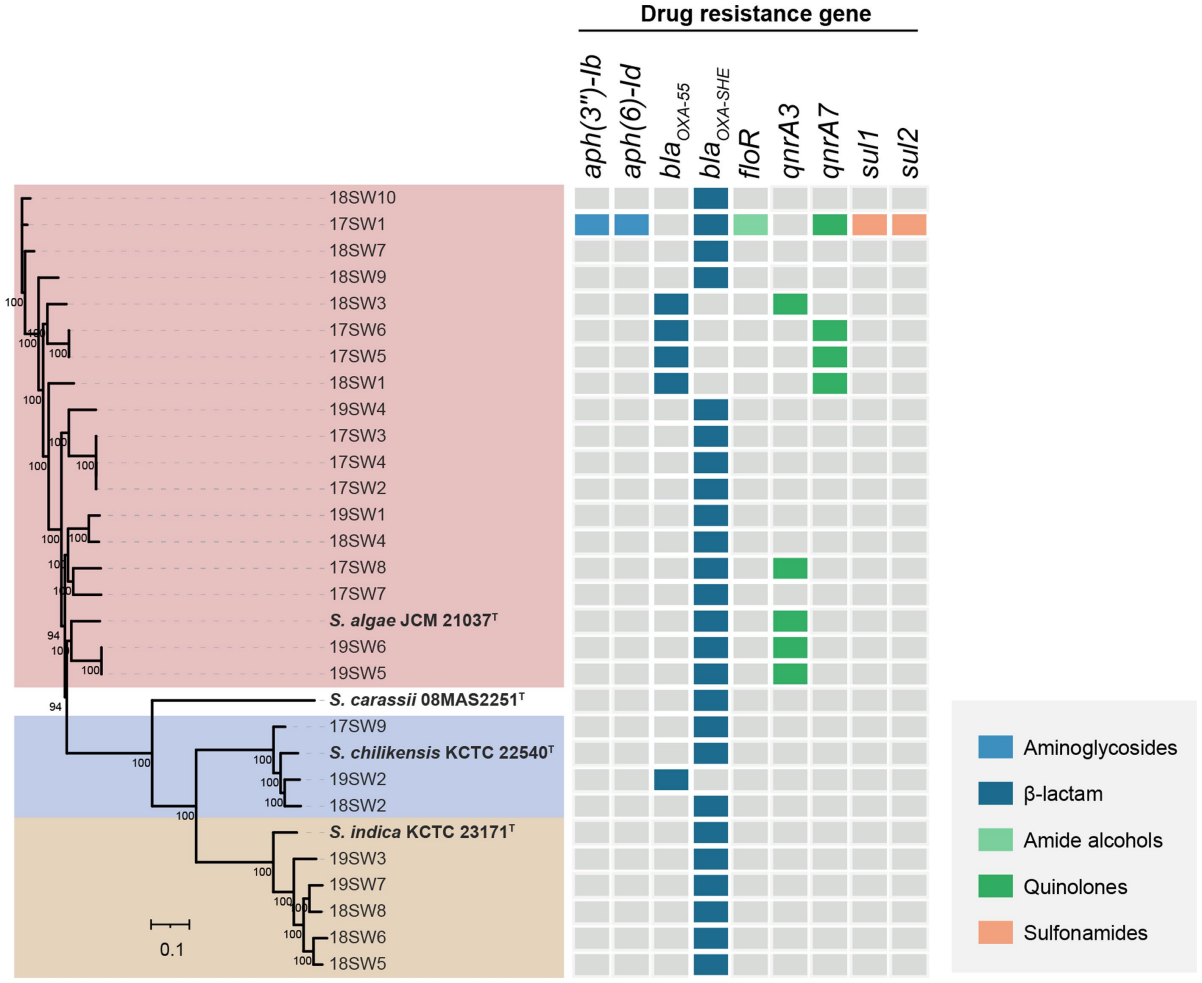
The heatmap of the antimicrobial resistance genes in 26 *Shewanella* strains. Gray color represents the presence of antimicrobial resistance gene and other colors represent the presence of different antimicrobial resistance genes.

A total of nine ARGs were identified by searching against the CARD (Figure 5). Only The strain 17SW1 was a multidrug-resistant stain, which carries the highest number of antimicrobial resistance genes (*aph(3″)-Ib*, *aph(6)-Id*, *bla_OXA-SHE_*, *floR*, *qnrA7*, *sul1*, *sul2*), indicating a capacity to resist to aminoglycosides, β-lactam, amide alcohols, quinolones, sulfonamides. The *bla_OXA_* gene was located in all strains with the major genotype of *bla_OXA-SHE_* (80.77%). Nine strains carried *qnr* with the most common genotype of *qnrA3*.

### 3.7 Antibiotic susceptibility of *Shewanella* spp.

**Figure 6:**
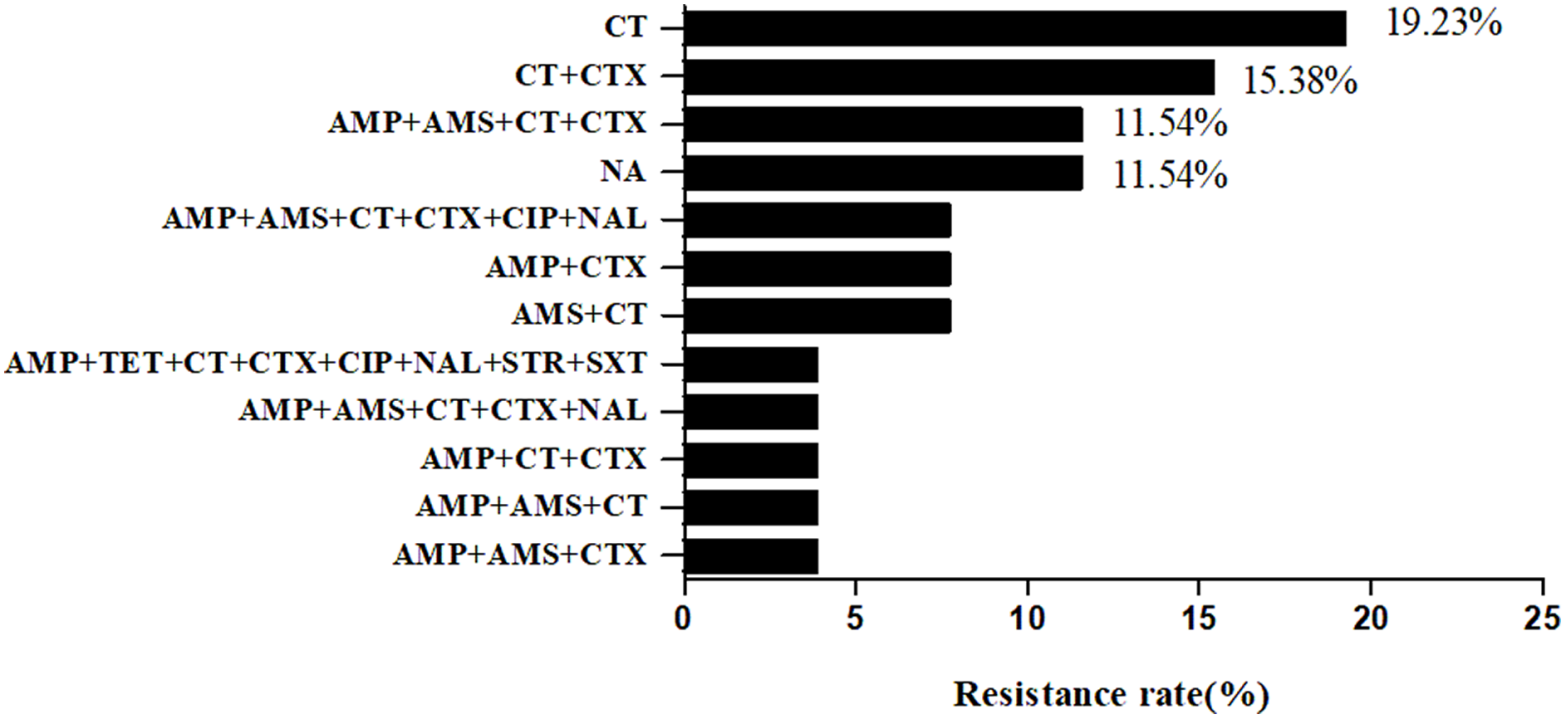
Antibiotic Susceptibility spectrum of 26 *Shewanella* strains. AMP, ampicillin; AMS, ampicillin-salbactam; TET, tetracycline; MEM, meropenem; CT, polymyxin E; ETP, Ertapenem; CZA, Ceftazidime/avibactam; TGC, Tigecycline; CTX, cefotaxime; CAZ, ceftazidime-avibactam; CIP, Ciprofloxacin; AZI, azithromycin; CHL, chloramphenicol; NAL, nalidixic acid; STR, streptomycin; SXT, selectrin; AMK, amikacin. NA, no resistance to all antibiotics.

The resistance rates of 26 strains to 17 antibiotics from high to low were polymyxin E (76.92%, 20/26), cefotaxime (53.85%, 14/26), ampicillin (50%, 13/26), ampicillin-salbactam (34.62%, 9/26), nalidixic acid (15.38%, 4/26), ciprofloxacin (11.54%, 3/26), selectrin (3.85%, 1/26), and tetracycline (3.85%, 1/26). None of the strains were resistant to ceftazidime/avibactam, ceftazidime-avibactam, tigecycline, meropenem, chloramphenicol, streptomycin, ertapenem, azithromycin, or amikacin. The rate of multidrug resistance was 38.46% (10/26). There were 12 types of drug resistance spectra (Figure 6), with the dominant spectrum being polymyxin E. The antimicrobial susceptibilities of the different strains are shown in Table 3.

**Table 3:** The resistance rate of different *Shewanella* strains.

	*S. algae* (%)	*S. chilikensis* (%)	*S. indica* (%)	*Shewanella* (%)
AMP	72.22	0	0	50.00
AMS	50.00	0	0	34.62
TET	5.56	0	0	3.85
MEM	0	0	0	0.00
CT	77.78	33.33	100	76.92
ETP	0	0	0	0.00
CZA	0	0	0	0.00
CTX	77.78	0	0	53.85
CAZ	0	0	0	0.00
CIP	16.67	0	0	11.54
CHL	0	0	0	0.00
NAL	22.22	0	0	15.38
SXT	5.56	0	0	3.85
AMK	0	0	0	0.00

AMP, ampicillin; AMS, ampicillin-salbactam; TET, tetracycline; MEM, meropenem; CT, polymyxin E; ETP, Ertapenem; CZA, Ceftazidime/avibactam; TGC, Tigecycline; CTX, cefotaxime; CAZ, ceftazidime-avibactam; CIP, Ciprofloxacin; AZI, azithromycin; CHL, chloramphenicol; NAL, nalidixic acid; STR, streptomycin; SXT, selectrin; AMK, amikacin.

## 4 Discussion

To date, more than 70 species of *Shewanella* have been identified.^2^ Several *Shewanella* species are opportunistic pathogens that cause infectious diseases in aquatic animals and humans. *Shewanella* has aroused widespread concern owing to the rapid increase in the number of identified species and the increasing reports of relevant clinical cases (Yu et al., 2022).

In this study, a long-term diarrheal disease-based surveillance program showed a detection rate of 2.36% for *Shewanella*. The epidemiological and clinical symptoms of *Shewanella* infection are similar to those of halophilic marine *Vibrio* species (Holt et al., 2005). *Shewanella* showed a clear seasonal distribution. Specifically, peak detection was concentrated in July and August (summer), whereas no detection occurred from December to April (the following year, winter), consistent with previous monitoring surveys. This may be because seafood circulation is frequent in the summer, with temperature and humidity facilitating *Shewanella* growth and foods being prone to cross-contamination, which leads to food-borne infections. A report from Anhui Province, China, demonstrated the isolation of *S. algae* from patients with food poisoning (Wang et al., 2013).

The Shewanella samples collected in this study included three main species: *S. algae* (69.23%), *S. indica* (19.23%), and *S. chilikensis* (11.54%). The allelic profiles of *Shewanella* isolates were classified into 22 STs. Among these, *S. algae* were classified into 16 STs, accounting for 66.7%. *S. indica* and *S. chilikensis* were classified into five and three STs, accounting for 8.8% and 7.4%, respectively. The dominant species isolated from clinical settings, such as blood, sputum, urine, and abdominal cavity effusion, were *S. algae* and *S. putrefaciens* (Yu et al., 2022). Moreover, *S. xiamenensis* has also been isolated from anal swab samples (Antonelli et al., 2015). *S. indica* and *S. chilikensis* are often isolated from seafood and cooked food (Wang et al., 2013), leading to an increased risk of foodborne diseases. In our study, *S. algae* was the dominant species detected in the genus *Shewanella*, which is highly associated with clinical diseases. Fang et al. (2019) showed that *S. algae*, *S. indica*, and *S. chilikensis* are clinically relevant with highly similar 16S rRNA sequences. Therefore, *S. indica* and *S. chilikensis* isolates may have been misidentified as *S. algae* in the molecular diagnosis. We sequenced the whole genome of 26 *Shewanella* isolates and performed higher-resolution MLST and ANI for the accurate identification of *Shewanella* species.

MLST is a common molecular typing method with high discriminatory power that has developed rapidly in recent years. MLST is suitable for both molecular epidemiology and evolution studies, as well as for international reference laboratories to establish a typing system for comparing strains worldwide (Perez-Losada et al., 2013; Floridia-Yapur et al., 2021). This study utilized seven previously described housekeeping genes to explore the genetic correlation of *Shewanella* isolates, and provide a scientific basis for studying the overall evolutionary structure and phylogenetic relationships of *Shewanella*. MLST classified the isolated strains of *Shewanella* into 22 STs, and the pathogen spectrum of *Shewanella* was rich and diverse, indicating that these strains have high genetic diversity. MLST is simple to use and can provide quick and easy comparisons between different laboratories. It has become a routine typing method for various bacteria, and it can be used for epidemiological monitoring and evolutionary research on *Shewanella*. MLST can be used as a molecular typing method for routine *Shewanella* surveillance, which should be increased to provide a scientific basis for the prevention and control of clinical infections.

Pan-genome analysis revealed approximately 3,000 core genomes from 18 *S. algae* strains. Core genome function annotation showed that the main functions were bacterial basal metabolism and biosynthesis, such as metabolism and energy production of various substances. This result is consistent with that of a core genome analysis of *S. algae* (Huang et al., 2022). The functions of auxiliary genomes, especially those of specific genes, are primarily related to DNA replication and repair, biofilm formation, and cell movement. Auxiliary genomes, represented by virulence islands and prophages, are one of the main driving forces of the genetic evolution and environmental adaptation of *S. algae* (Huang et al., 2022). *S. algae* has high intra-species diversity in population structure and genetic evolution, which contributes to the evolution of its pathogenic mechanism and environmental adaptability (Janda and Abbott, 2014). By exploring the relationship between the sequence diversity of virulence factors and different *Shewanella* strains, this study revealed differences in the composition of virulence genes among *S. algae*, *S. indica*, and *S. chilikensis*, indicating that the virulence spectra of the different *Shewanella*e species were different. However, all three *Shewanella* species were isolated from clinical samples, indicating that *Shewanella* pathogenesis may be influenced by multiple factors and may be the result of complex interactions between the host and the environment. This suggests that different *Shewanella* strains are initiation factors, rather than determining factors, for human diseases, and that the pathogenic process may result from the synergistic effect of multiple factors (Yu et al., 2022).

Cerbino et al. (2023) performed virulome analysis on *Shewanella* spp. They put forward that there is a correlation between the VAS T6SS and the *S. algae* lineage. T6SS may be a key virulence system that contributes to *S. algae* virulence. Furthermore, irgA (iron-regulated adhesin), lasB (elastase), and zmp1 (Zn-metalloprotease) homologs were detected mostly in *S. algae* and *S. xiamenensis*. Chemosensory and c-di-GMP signal transduction systems integrate environmental stimuli to modulate gene expression, including the switch from a planktonic to sessile lifestyle and pathogenicity. Alberto et al. provide an inventory of the c-di-GMP turnover proteome and chemosensory networks of *S. algae*. *S. algae* strains encoded 61–67 c-di-GMP turnover proteins and 28–31 chemoreceptors, placing *S.algae* near the top in terms of these signaling capacities per Mbp of genome (Cerbino et al., 2023).

Differences in antibiotic susceptibilities of clinical *Shewanella* isolates have been reported. However, they are usually susceptible to third- and fourth-generation cephalosporins, β-lactamase inhibitor combinations, and quinolones (Vignier et al., 2013; Yousfi et al., 2017a). *Shewanella* isolates in this study showed resistance to ampicillin-sulbactam (β-lactamase inhibitor combination), cefotaxime, ciprofloxacin (quinolones), and nalidixic acid (quinolones). This suggests that an increased risk of drug resistance in *Shewanella* may contribute to clinical treatment failure. The high resistance rate of *S. algae* to ampicillin in this study (72.22%, 13/18), despite its large variations in practice (Holt et al., 2005), suggests that *S. algae* drug resistance is more serious in this region and should be continuously monitored. Moreover, 76.92% of *Shewanella* isolates were resistant to polymyxin E in this study. Related studies confirmed that *Shewanella* spp. isolated from neonatal patients with sepsis (Charles et al., 2015) and wounds of patients bitten by cobras (Liu et al., 2014) were resistant to polymyxin E, which may be related to the different sources and regions of strain isolation. Surprisingly, the strains exhibited a polymyxin resistance phenotype yet no determinants were detected. The molecular mechanisms of polymyxin resistance has been characterized, including specific modification of outer membrane porins, reductions in the overall negative charge of the LPS, overexpression of efflux pump systems, and overproduction of capsule polysaccharide (Bialvaei and Samadi Kafil, 2015). Polymyxin resistance in Gram-negative bacteria is commonly due to decreased binding to the bacterial outer membrane because of lipopolysaccharide remodeling that is caused by changes in *PhoPQ* and *PmrAB*, both two-component regulatory systems (Zavascki et al., 2007; Lopez-Rojas et al., 2011). Acquired polymyxin resistance most often mediated by replacement of lipid A by addition of 4-amino-4-deoxy-L-arabinose (L-Ara4N) and/or phosphoethanolamine (PEtn) (Moffatt et al., 2010). Because of this effect, which is mediated by *pmrC* and necessitates the products of the *ugd* and *pbg* loci and ethanolamine, these alterations eliminate negative charges, decreasing the affinity of LPS and boosting resistance to polymyxins (Beceiro et al., 2011). In addition, a non-specific mechanism for the tolerance of polymyxin was shown to be up-regulation of the *MexAB-OprM* efflux pump (Beceiro et al., 2011). The genetic mechanisms underlying colistin resistance in *Shewanella* are not well understood. Huang et al. elaborated a combination of three mutations (PmrB 451, PmrE168, PmrH292) that were strongly associated with colistin resistance in *S. algae* (Beceiro et al., 2011). Therefore, the mechanism of polymycin resistance in *Shewanella* needs to be deeply explored in combination with molecular experiments and genomic information.

Compared to *Vibrio parahaemolyticus* isolated from patients with diarrhea in Beijing, China, which is widely distributed in the marine environment and is halophilic, *Shewanella* spp. showed a high level of resistance to various antibiotics. In addition, one *S. algae* isolate in this study also showed combined resistance to ampicillin + tetracycline + polymyxin E + cefotaxime + ciprofloxacin + nalidixic acid + streptomycin + cotrimoxazole, suggesting that *S. algae* may be prone to carrying resistance elements that can become carriers of resistance genes or even undergo horizontal transfer. In 2017, *S. xiamenensis* isolated from hospital wastewater had several drug resistance genes that were resistant to trimethoprim, aminoglycosides, quaternary ammonium compounds, β-lactams, chloramphenicol, sulfonamides, and tetracyclines (Yousfi et al., 2017b). Due to the widespread use of antibiotics, *Shewanella* is at risk of becoming a superbacterium.

## Data availability statement

The data presented in this study are deposited in the NCBI database under accession number PRJNA1015006.

## Ethics statement

The studies involving humans were approved by The Ethical committee of Beijing Center for Disease Prevention and Control. The studies were conducted in accordance with the local legislation and institutional requirements. The human samples used in this study were acquired from primarily isolated as part of our previous study for which ethical approval was obtained. Written informed consent for participation was not required from the participants or the participants’ legal guardians/next of kin in accordance with the national legislation and institutional requirements.

## Author contributions

YK: Writing – original draft, Data curation, Formal analysis, Visualization, Validation. KY: Data curation, Writing – original draft, Software. ZH: Data curation, Writing – original draft, Software. BP: Methodology, Writing – review & editing. SL: Writing – original draft, Investigation, Validation. TP: Writing – original draft, Investigation, Validation. YL: Writing – review & editing, Conceptualization, Methodology, Supervision. DW: Writing – review & editing, Conceptualization, Funding acquisition, Resources.

## Footnotes

^1^ https://rast.nmpdr.org/

^2^ http://www.bacterio.net/Shewanella.html
